# An Unusual Benign Cause of an Alarming Finding on Chest-X ray: A Case Report of Widened Mediastinum due to Rare Congenital Abnormality (Azygos Vein Continuation of Inferior Vena Cava)

**DOI:** 10.1155/2019/3457495

**Published:** 2019-12-09

**Authors:** Michael Hanna, Ghada Elshimy, Medhat Ismail, Mourad Ismail

**Affiliations:** ^1^Department of Medicine and Pulmonary Critical Care, New York Medical College at Saint Joseph's University Medical Center, 703 Main St., Paterson, NJ, USA; ^2^Department of Endocrinology, Diabetes and Metabolism, University of Arizona College of Medicine, 1111 E McDowell Road, Phoenix, AZ, USA

## Abstract

Acute widened mediastinum is an alarming finding. It has many possible differential diagnoses; aortic dissection (AD) is considered one that carries catastrophic outcomes. AD is relatively uncommon; it requires early and accurate diagnosis and treatment for better patient survival. However, acute mediastinal widening also can be present in more benign conditions. We report a case of a 50-year-old African American female with postoperative shortness of breath; initial imaging studies revealed an acute widened mediastinum, but on further management with diuresis and follow-up imaging, she was diagnosed with azygous vein continuation of the Inferior vena cava (IVC). This is considered as a rare benign cause of wide mediastinum. Clinicians must be aware of the presence of such a benign cause when dealing with acute wide mediastinum.

## 1. Introduction

Widened mediastinum is defined as mediastinum with a measured width greater than 6 cm on an upright posterior–anterior chest radiograph (CXR) or 8 cm on supine anterior–posterior chest film [1]. Multiple etiologies can be identified including traumatic and nontraumatic causes; most of them require an urgent management to improve patients' survival. However, benign causes must be also included in the differential diagnosis especially if the patients are asymptomatic. Congenital anomalies of the IVC are considered one of the benign causes [2]. We report a rare congenital anomaly of the IVC in a patient with wide mediastinum.

## 2. Case Presentation

**Figure 1:**
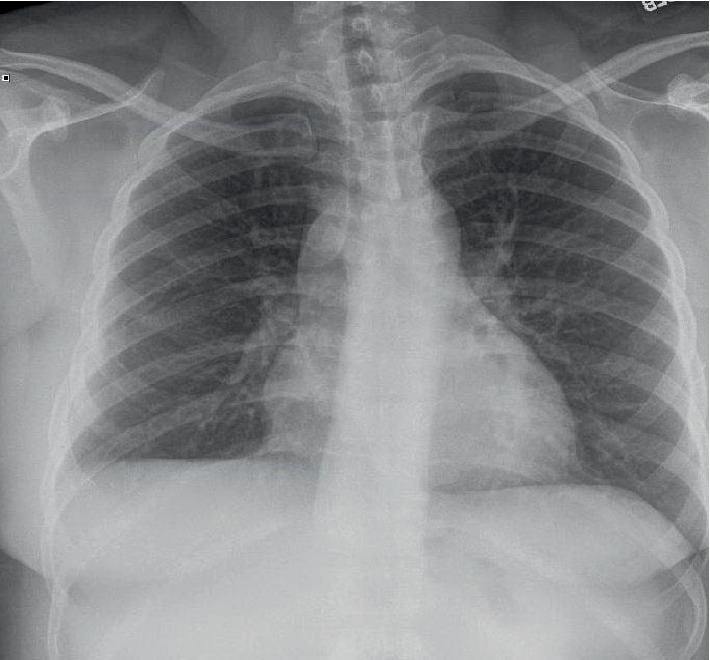
Preoperative upright postero-anterior CXR demonstrating normal width mediastinum.

**Figure 2:**
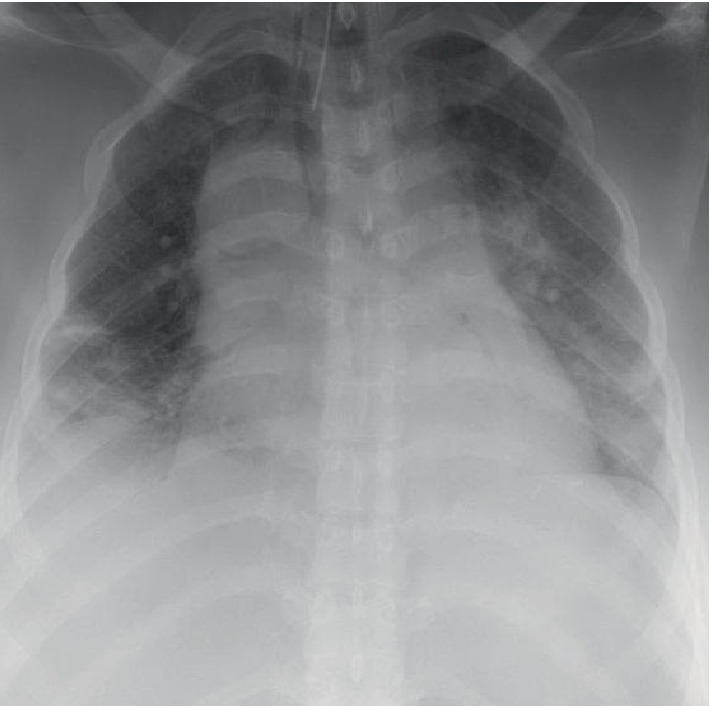
Postoperative day 0 supine antero-posterior CXR demonstrating wide mediastinum.

**Figure 3:**
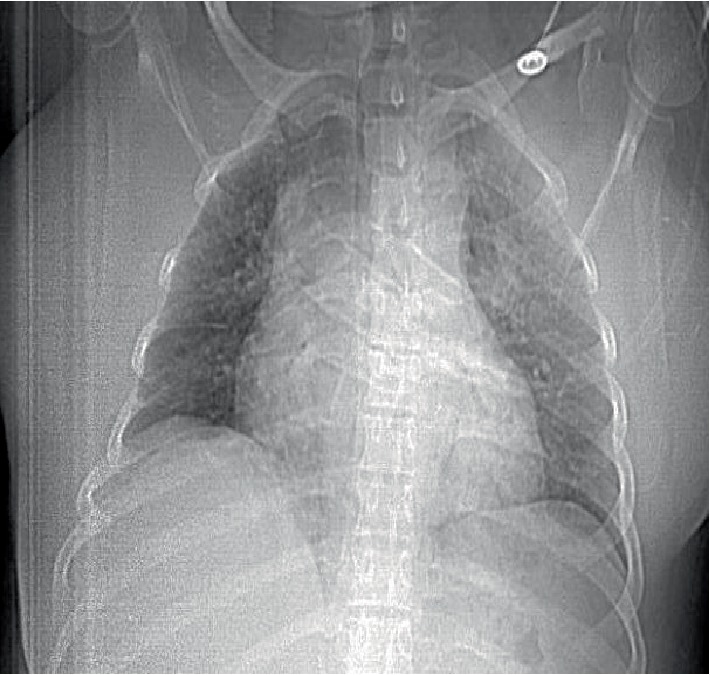
Postoperative day 0 CAT scan scout view demonstrating wide mediastinum.

**Figure 4:**
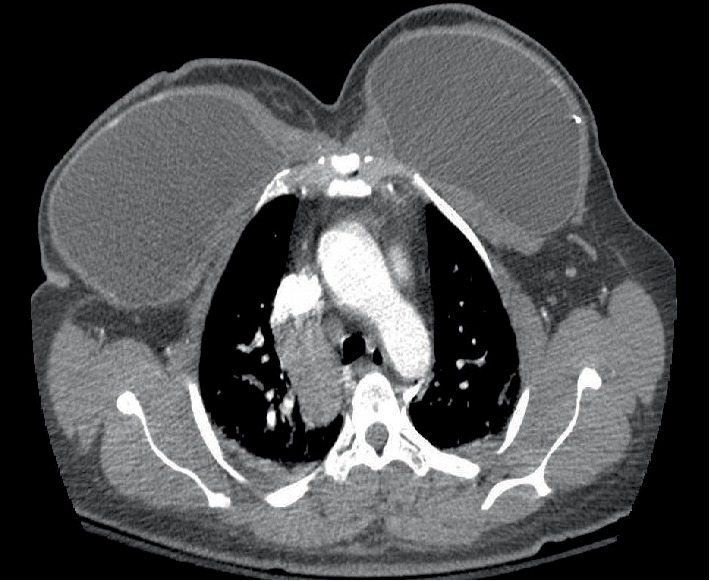
Postoperative day 0 CAT scan axial cut demonstrating widening of the mediastinum due to enlarged azygos arch. Arrow is pointing at azygos arch.

**Figure 5:**
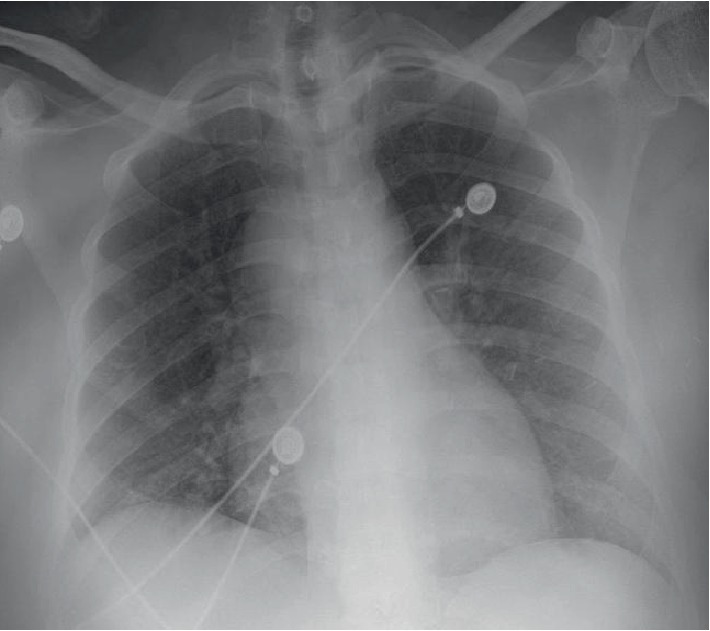
Postoperative day 1 antero-posterior CXR semi-erect position demonstrating normal width mediastinum after diuresis.

The patient is a 50-year-old African-American female with past medical history of essential hypertension, diabetes mellitus type 2, diabetic peripheral neuropathy, hyperlipidemia, chronic kidney disease stage 3, gastroesophageal reflux disease, and bilateral breast augmentation surgery done 3 years ago without pre or postoperative complications. Patient never smoked and she stated that she consumes alcohol only socially. She denied any recreational drug use. She presented to our hospital for an elective anterior lumbar interbody fusion surgery for spinal stenosis. The patient had an uneventful surgical procedure, during which she received 1500 cc of crystalloids and 2 doses of 5% albumin 25 grams each. Post-operatively, while patient is in post anesthesia care unit, she developed sudden onset of shortness of breath with mild hypoxemia. Patient did not complain of substernal chest pain, palpitations, hemoptysis, wheezing, leg pain, or swelling. Vital signs included a temperature of 98.1 F, blood pressure (BP) of 164/86 mmHg, respiratory rate of 16 breaths/min, regular pulse with heart rate (HR) of 96 beats/min, and oxygen saturation of 88% on room air. Physical examination revealed an obese female in mild distress but non labored breathing. The neck exam showed midline trachea with no jugular venous distension, carotid bruits, or thyromegaly. Lung exam disclosed fine inspiratory crackles at the bases bilaterally with no expiratory wheezing. The rest of the physical exam was unremarkable. Electrocardiogram (EKG) showed normal sinus rhythm with nonspecific ST deviation. Laboratory findings were as follows: white blood cell 13,700/*µ*L, hemoglobin 9.3 g/dL, platelet count 293 K/mm, creatinine 1.47 mg/dL (GFR = 46), bicarbonate 24 mg/dL, baseline creatinine 1.72 mg/dL. A plain CXR was done. When it was compared to a study done 10 days earlier (Figure 1), it demonstrated widening of the mediastinum (Figure 2). Since AD was a major concern, urgent measures to lower BP and control the HR were required. Labetalol drip was started immediately at 1 mg/min. A follow-up emergent chest Computed Tomography Angiography scan (CTA) was performed (Figures 3 and 4). It showed widening of the mediastinum due to enlarged azygos arch, and azygos vein continuing in retro-crural space with azygos continuation of the IVC. No hilar lymphadenopathy or AD was noted. A diagnosis of azygos vein continuation of the IVC was made. Furosemide 40 mg intravenous was given for the suspected pulmonary edema and fluid overload. The patient's dyspnea improved with significant diuresis of 2000 mL clear urine. A repeat plain CXR the next day showed normal mediastinal width (Figure 5). The acute widening of the mediastinum was presumed to be secondary to fluid overload which resulted in engorged azygos vein which presented as a widened mediastinum.

## 3. Discussion

Inferior vena cava is the result of unifying four segments: hepatic, suprarenal, renal, and infrarenal, after a complex embryogenic process. The whole venous system starts from three pairs of primitive veins: common cardinal, umbilical, and vitelline veins join together in the sinus venosus. The common cardinal vein is formed by the union of the anterior and posterior cardinal veins. Anastomosis of the vitelline pair of veins develops and forms the hepatic venous system including the hepatic segment of the IVC [3]. Subcardinal veins develop later with significant anastomosis between each other and with the posterior cardinal veins, to form the gonadal veins, and the suprarenal segment of the IVC [4]. A pair of supracardinal veins then develops and anastomoses with the posterior cardinal veins, and the subcardinal veins to form the subsupracardinal anastomosis. Subsupracardinal anastomosis gives rise to the renal veins and the renal segment of the IVC. Supracardinal veins form the azygos vein and the infrarenal segment of the IVC [3, 5].

Azygos continuation of the IVC exists when there is an embryogenic failure to form the right subcardinal–hepatic anastomosis, in such cases, the blood drains from both kidneys through the renal segment of the IVC and passes posterior to the diaphragm entering the thorax as the azygos vein. The azygos vein terminates in the superior vena cava (SVC) similar to the normal pattern [3].

Azygos vein is generally formed at the 1^st^ lumbar vertebra level from the right ascending lumbar vein and the right subcostal vein, and then travels cranially on the right side of the vertebral column to open into the SVC at the level of the 5^th^ and 6^th^ thoracic vertebrae. Veins that drain into the Azygos are the right superior intercostal vein, 5^th^–11^th^right posterior intercostal veins, hemiazygos vein, accessory hemiazygos vein, pericardial, bronchial, and esophageal veins. The Azygos provides collateral venous circulation between the SVC and the IVC with two ways shunt since it has few to no valves [6]. As such, the Azygos vein has a significant clinical role in cases of increased venous pressure.

Microvascular fluids under physiological conditions are under hydrostatic pressure of filtration (which is composed of hydrostatic pressure and peri-microvascular pressure of elastic return), opposed by colloid-osmotic pressure, resulting in a final filtration pressure of about 1 mmHg. This causes continuous leak of water and solutes out of the micro vessels. In the lung, the lymph system prevents pulmonary edema by evacuating the leaking water and solutes. Pulmonary edema might develop in case of elevated hydrostatic pressure, lessened colloid-osmotic plasmatic pressure or a capillary injury. The Azygos vein and the vascular pedicle size on CXR correspond to the intravascular fluid status. Fluid overload results in the increase in the width of the azygos and the vascular pedicle. Studies showed an increase of 0.5 cm of the vascular pedicle corresponds to 1 L increase of circulating fluid [7, 8], while Milne and associates [7] proposed that the principle explanation behind expansion of the azygos vein is the increase in the mean right cardiac chamber pressure. This phenomenon can be observed in congestive heart failure or fluid overload where there is an expansion of the azygos vein. In cases where there is azygos vein continuation of the IVC, the azygos vein enlargement in the presence of fluid overload will be significantly amplified, and will masquerade as widened mediastinum [9].

Literature described IVC abnormal anatomies and showed 14 different variants, in some cases more than one variant can coexist [3, 9, 10]. However, azygos vein continuation of IVC is a rare variant accounts for 0.6% of the cases [8]. Diagnosis of the IVC anomalies is primarily made by imaging. Many reported cases were incidental findings during prenatal screening ultrasound [11]. Advanced imaging modalities such as CAT scan with intravenous contrast or Magnetic resonance angiogram (MRA) can be utilized for further delineation of anatomy. Experts prefer Multidetector Computerized Tomography (MDCT) as a fast, reliable diagnostic tool for IVC congenital vascular anomalies since it is less invasive than conventional angiography with a clear identification of thoraco-abdominal vascular structures [9].

Although the azygos continuation of the IVC has been described in the literature, our case is unique, since it documents the effect of the intravascular volume expansion on the mediastinal width in a patient with azygos continuation of the IVC. In our case, an acute wide mediastinum on CXR, triggered the alarm for further imaging to define the cause, and it unmasked the anomaly. Then, more interestingly, adequate diuresis restored a normal CXR. That gives a better understanding of such a situation, and helps to save other asymptomatic patients, with a known anomaly, in similar clinical scenarios from harmful investigations to rule out other urgent causes of acute wide mediastinum, such as: aortic aneurysm, AD, aortic rupture, esophageal rupture, mediastinitis.

## 4. Conclusion

This case shows the effect of the intravascular volume expansion on the mediastinal width in a patient with azygos continuation of the IVC, and provides an educational resource for better understanding of such a cause of an acute widened mediastinum.

## Abbreviations

EKG: Electrocardiogram
BP: Blood pressure
CTA: Chest computed tomography angiography scan
CXR: Chest radiograph
MRA: Magnetic resonance angiogram
MDCT: Multidetector computerized tomography
IVC: Inferior vena cava
SVC: Superior vena cava
HR: Heart rate.
